# Total hip arthroplasty, through the direct anterior approach, for a femoral neck fracture of ipsilateral transfemoral amputee: a case report

**DOI:** 10.3389/fsurg.2025.1556599

**Published:** 2025-03-18

**Authors:** Xiangde Zhao, Gangliang Wang, Yukang Wang, Jian Chen

**Affiliations:** ^1^ Department of Orthopaedic Surgery, Sir Run Run Shaw Hospital, Zhejiang University School of Medicine, Hangzhou, Zhejiang, China; ^2^ Key Laboratory of Mechanism Research and Precision Repair of Orthopaedic Trauma and Aging Diseases of Zhejiang Province, Hangzhou, Zhejiang, China; ^3^ Department of Orthopaedic Surgery, Wuyi County First People’s Hospital, Jinhua, China

**Keywords:** total hip arthroplasty, the direct anterior approach, femoral neck fracture, ipsilateral transfemoral amputation, osteoporosis

## Abstract

**Objective:**

Femoral neck fractures in transfemoral amputees are rare, and their management remains controversial, particularly in individuals with short residual limb lengths. Traditional approaches to total hip arthroplasty (THA), such as the anterolateral, Hardinge, and posterior methods, present significant challenges, including difficulties in residual limb manipulation, femoral exposure, and postoperative swelling. The direct anterior approach (DAA), a minimally invasive technique, has not been previously reported for THA in this patient population. This case report aims to evaluate the feasibility and outcomes of the DAA for THA in a transfemoral amputee with a femoral neck fracture.

**Methods:**

We present the case of a 64-year-old Han male with an ipsilateral transfemoral amputation who sustained a femoral neck fracture. The patient underwent THA using the direct anterior approach. The DAA was selected due to its minimally invasive nature and ability to navigate through the intermuscular interval, which facilitated improved manipulation of the residual limb and easier exposure of the femoral side. Standard surgical protocols were followed, and postoperative care included monitoring for complications such as infection, thrombosis, and dislocation.

**Results:**

The patient's postoperative recovery was uneventful, with no signs of infection, thrombosis, dislocation, or other complications. Notably, there was no significant residual limb swelling, which was attributed to the minimally invasive nature of the DAA. The patient achieved unrestricted hip mobility without postural restrictions and attained a Harris Hip Score of 84.78 at follow-up, indicating a favorable functional outcome.

**Conclusions:**

This case demonstrates the efficacy and safety of the direct anterior approach for total hip arthroplasty in patients with high above-knee amputations. The DAA effectively addresses many challenges associated with traditional approaches, such as residual limb manipulation, femoral exposure, and postoperative swelling. These findings suggest that the DAA is a viable alternative for managing femoral neck fractures in transfemoral amputees, warranting further investigation in larger studies to validate its broader applicability.

## Introduction

Globally, while non-amputated femoral neck fractures are very common with a consensus on their treatment, femoral neck fractures in transfemoral amputees are rare and lack consensus regarding their management (1). In the United States alone, approximately 30,000–40,000 individuals undergo lower limb amputations annually (2). Nevertheless, reports on femoral neck fractures in amputees remain scarce (3). Therefore, it is crucial to devote adequate attention to the management and treatment options for femoral neck fractures occurring on the same side as the amputation.

Various surgical approaches have been explored for patients with ipsilateral transfemoral amputation. Michael Leonard et al. reported a case of total hip arthroplasty (THA) performed using the antero-lateral approach. The authors inserted a 5 mm Steinman pin in the distal end of the stump to facilitate stump control, although this technique may potentially increase the risk of infection and fracture due to reduced bone mass (4). Hassan Boussakri et al. described a case of hip arthroplasty in a patient with transfemoral amputation utilizing Hardinge's approach and employing a bone forceps under the lesser trochanter for improved manipulation of the stump, which could potentially lead to increased surgical trauma (5). Owen J. Diamond et al. presented a case report on THA following an ipsilateral above knee amputation through the posterior approach, where they placed a Steinman pin in the great trochanter to enhance leverage on the femur and achieve better exposure on its femoral side; however, this technique also carries an increased risk of fracture (6). Additionally, they reported postoperative stump swelling as another challenge encountered (4).

The direct anterior approach was initially described by Hueter and subsequently by Smith-Petersen et al. and Judet (7). DAA accesses the hip joint through the interval between the tensor fascia latae and the rectus femoris, which is associated with a decreased risk of dislocation, accelerated recovery, reduced pain, and fewer surgical complications (8). Therefore, DAA is particularly beneficial for patients with ipsilateral transfemoral amputation.

## Case presentation

**Figure 1:**
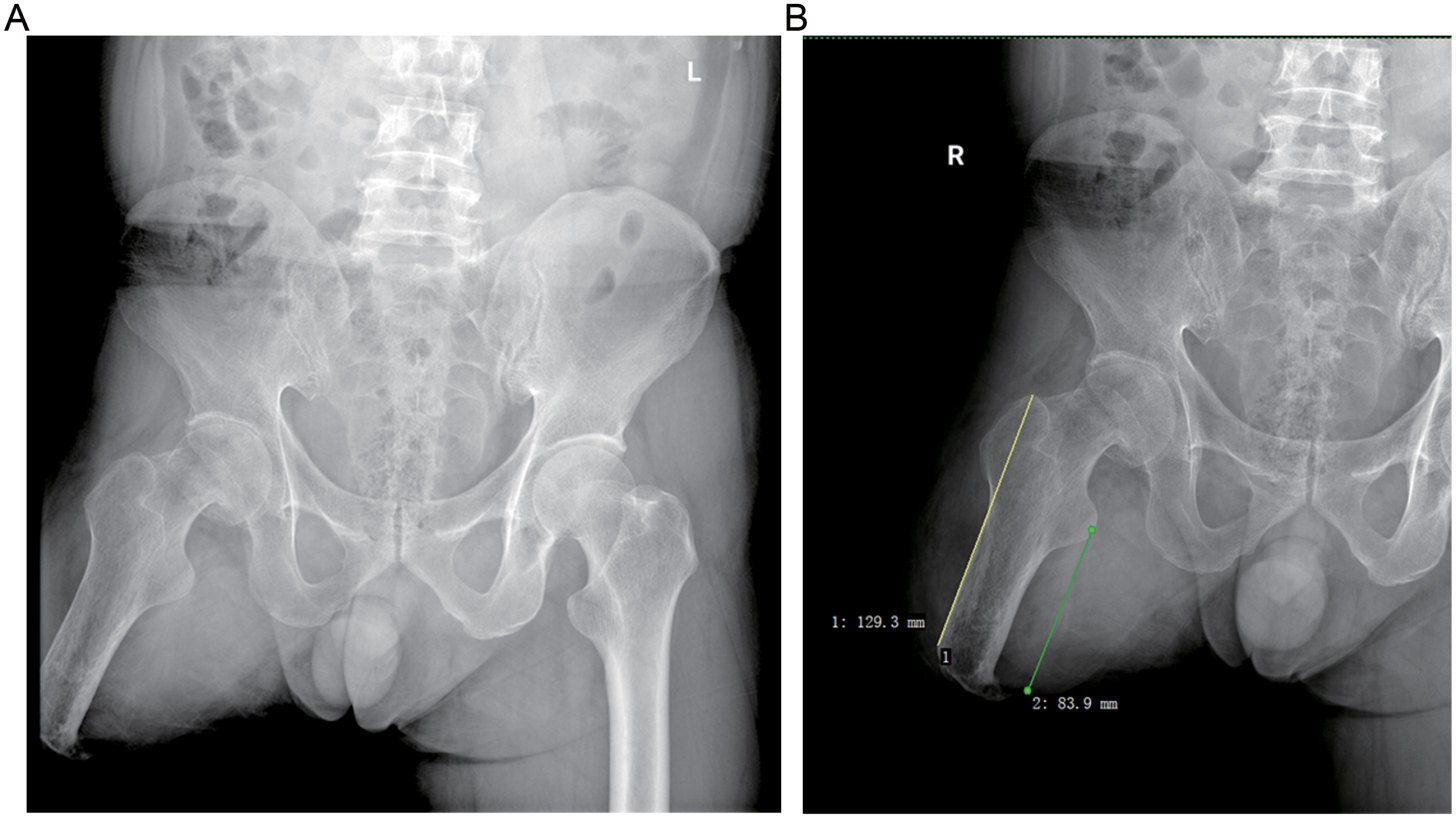
Preoperative radiograph of the patient, showing a femoral neck fracture and signs of bone mass reduction of the right hip. **(A)** Anteroposterior pelvic x-ray. **(B)** Anteroposterior x-ray of the right hip joint.

**Figure 2:**
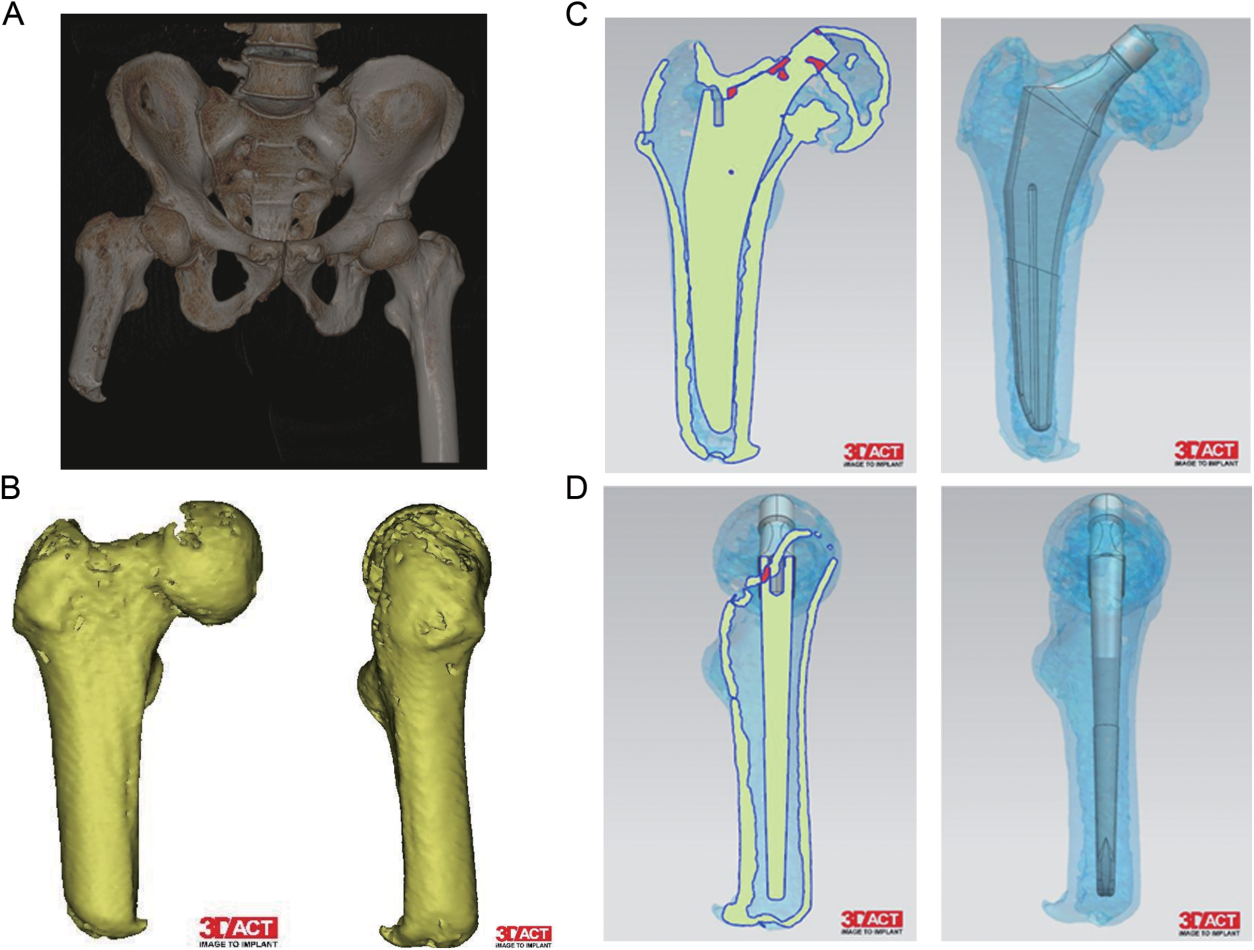
Preoperative CT 3D reconstruction and preoperative planning image. **(A)** CT 3D reconstruction of the pelvic. **(B)** CT 3D reconstruction of the right femur. **(C,D)** Preoperative planning images of the femur.

The patient, a 64-year-old Han male, was admitted to the hospital due to right hip pain following a fall that occurred half a day ago. He had undergone right femoral amputation 30 years ago as a result of a work-related injury and has been using a prosthetic limb for walking or driving ever since. Additionally, he has had hypertension for three years, and his blood pressure has been well controlled. Upon admission, the patient presented with significant fear of moving his right hip and exhibited obvious amyotrophy in the distal region of his right thigh. Imaging data revealed a Garden III type femoral neck fracture on the right side with decreased bone density (Figure 1). The residual limb length was 129 mm from the greater trochanter to the distal (Figure 1). After thorough discussion regarding available treatment options including internal fixation, joint replacement, or conservative management, taking into consideration factors such as the patient's age and high activity level, joint replacement was chosen as the most suitable course of action. A CT scan of the hip was performed to further assess bone mass and facilitate preoperative planning for prosthesis selection (Figure 2).

As the direct anterior approach (DAA) total hip arthroplasty offers the advantages of being less invasive, faster recovery, and reduced pain, DAA was employed for joint replacement, and a biocompatible prosthesis was selected based on preoperative planning. The patient assumed a supine position on the operating table under general anesthesia, and an approximately 10 cm long incision was made on the anterior-lateral aspect of the right hip. Utilizing the DAA to accesses the hip joint through the interval between the tensor fascia latae and the rectus femoris, the joint capsule was incised, followed by femoral neck osteotomy to remove the femoral head and partial neck. Subsequently, a 48 mm acetabular cup (iKang, China) with an XPE liner was inserted. To expose the proximal femur adequately, an assistant internally rotated and adducted the residual limb while applying pressure to its distal stump. To prevent intraoperative calcar fracture during femoral prosthesis insertion, wires were wrapped around the femoral shaft due to osteoporosis of the residual femur. The chosen femoral stem (iKang, China) featured a proximal porous coating along with a distal non-porous coating. For secure fixation, bone cement was applied at the distal end of the femoral canal prior to inserting both the femoral stem and a 32 mm ceramic head (Delta, America). No intraoperative fractures occurred during this procedure. Postoperatively, antibiotics were administered as prophylaxis against infection while anticoagulant therapy was initiated.

**Figure 3:**
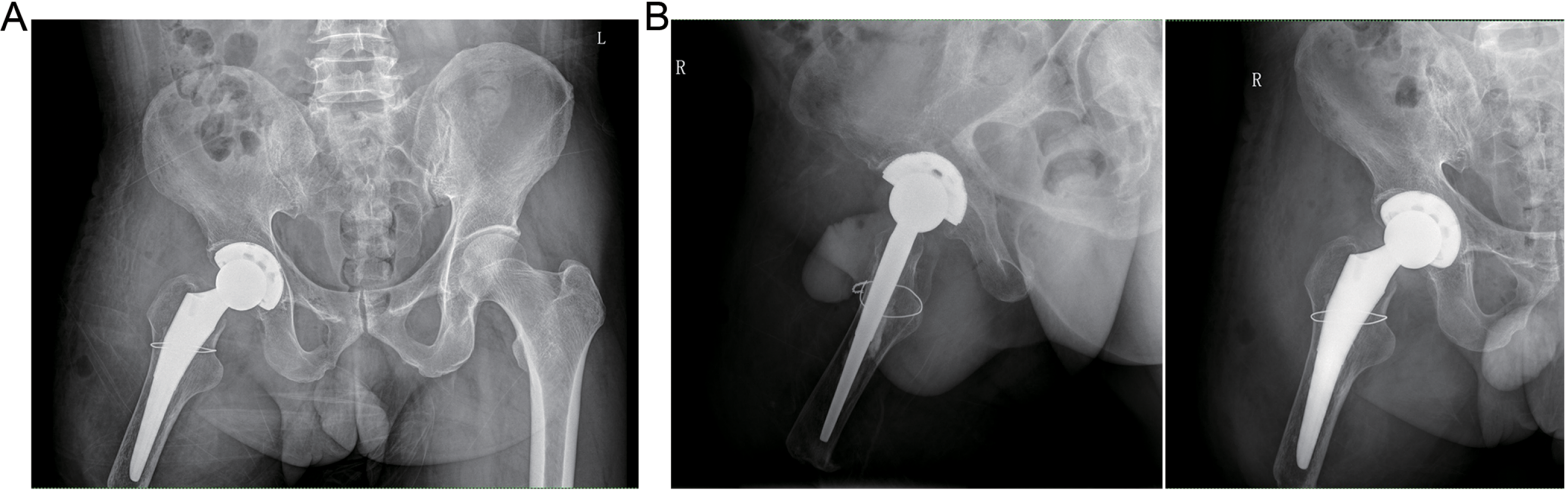
Postoperative radiograph of the patient at 1 day. **(A)** Anteroposterior pelvic x-ray. **(B)** Anteroposterior x-ray of the right hip joint and lateral x-ray of the right femoral neck.

**Figure 4:**
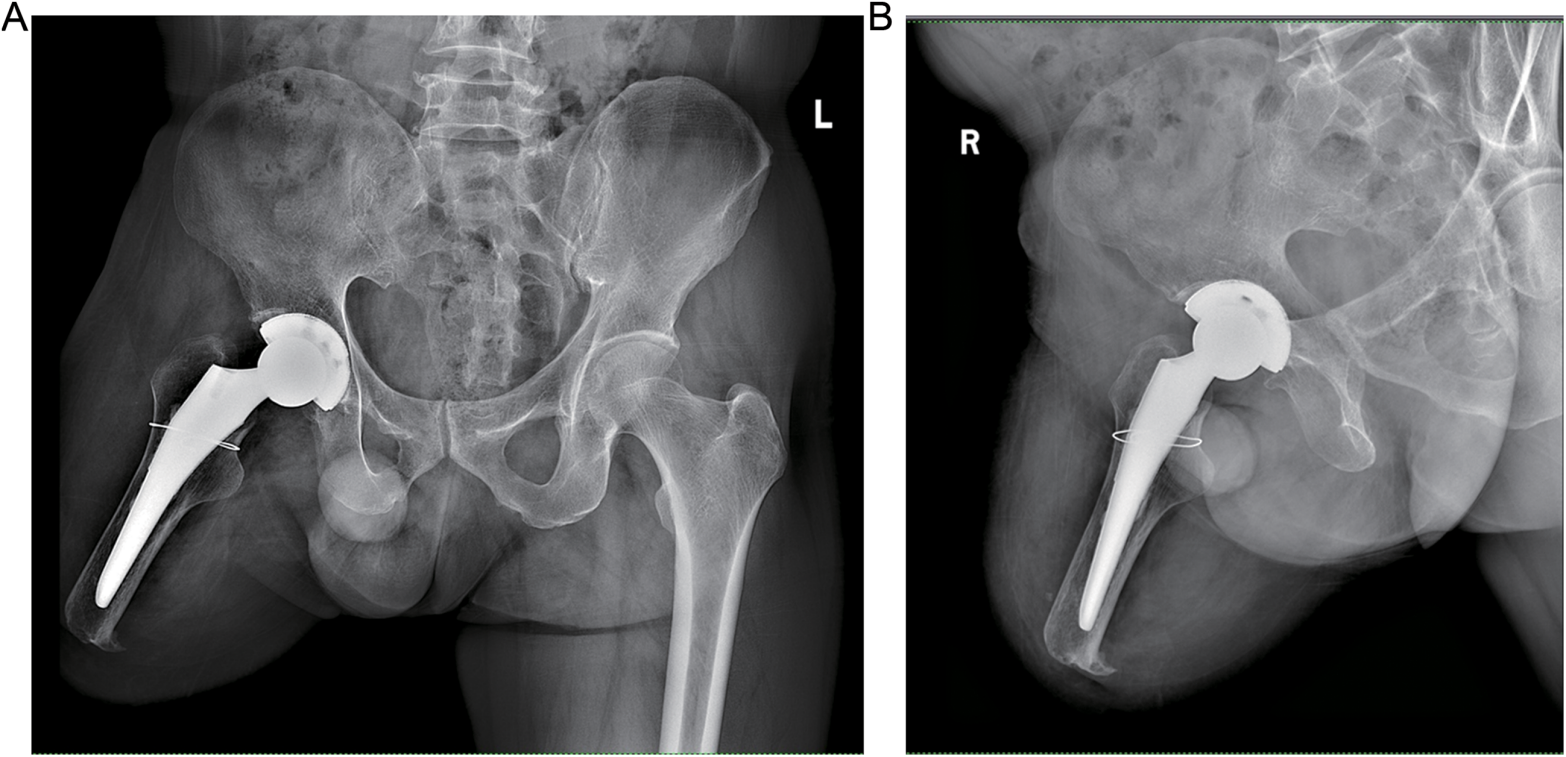
Postoperative radiograph of the patient at 5 months. **(A)** Anteroposterior pelvic x-ray. **(B)** Anteroposterior x-ray of the right hip joint.

The patient exhibited a smooth postoperative recovery without any indications of infection, thrombosis, dislocation, or other complications (Figure 3). No evident residual limb swelling was observed. Throughout the hospitalization period, the patient had unrestricted movement of their right hip and was permitted to sit and actively engage in both passive and active rehabilitation exercises. Discharge took place six days after surgery. Suture removal occurred two weeks postoperatively while continuing with rehabilitation exercises. At the six-month follow-up assessment, the condition of the residual limb remained favorable with no activity-related pain reported by the patient. Imaging data revealed no signs of bone resorption in either the acetabular cup or femoral prosthesis (Figure 4). The Harris Hip Score for this patient was recorded as 84.78.

## Discussion

Although the treatment and prognosis of femoral neck fractures have been extensively documented, there is a paucity of literature on total hip arthroplasty for femoral neck fractures or osteoarthritis in patients with ipsilateral femoral amputation (3–6, 9). Given the shorter residual limb length and reduced bone mass in patients with femoral amputation, total hip arthroplasty presents numerous challenges (6, 10). In order to dislocate the femoral head and facilitate better manipulation of the stump, Steinman or bone forceps are employed in the antero-lateral approach, Hardinge's approach, and posterior approach (4–6). However, these techniques not only increase surgical trauma but also pose risks such as infection and intraoperative fracture due to significantly reduced femoral bone density on the amputated side. Additionally, postoperative residual limb swelling warrants attention as it can impede implant installation (4).

The direct anterior approach has been associated with a reduced risk of dislocation, faster recovery, less pain, and fewer surgical complications (8). However, there have been no reported cases of DAA for femoral neck fractures in ipsilateral transfemoral amputees. Several factors should be considered when selecting the surgical approach. In this particular case, the residual limb length was 129 mm from the greater trochanter to the distal end with significant bone mass loss due to disuse. Considering the patient's age and high activity level, we opted for the direct anterior approach. Preoperative evaluation of imaging data must include measuring the full length of the residual limb to guide prosthesis selection. If there is a short residual limb or significant bone loss, a CT scan can be performed to assess bone mass and aid in preoperative planning for prosthesis selection. Short shank femoral prostheses are often used in such patients, which is stabilized early by proximal fixation and long-term by bone insertion. As For patients with osteoporosis, there is an increased risk of intraoperative calcar fracture during femoral prosthesis implantation (11, 12). To mitigate this risk, we used cerclage wires preoperatively to reduce calcar fracture incidence. In order to expose the proximal femur using traditional DAA technique requires flexing the bed and moving the femur into adduction and external rotation position; however, in this case where thigh amyotrophy was present, we were able to utilize buttock as a fulcrum by adducting and pressing on distal stump for exposing proximal femur instead. As our chosen femoral prosthesis had no coating at its distal end stem portion, bone cement was applied for achieving secure early fixation which guarantee the proximal bone insertion. To our knowledge, this is first time reporting use of DAA approach in treating such ipsilateral transfemoral amputee's femoral neck fracture.

Amputees' residual limbs often exhibit muscle atrophy and soft tissue laxity, which, when combined with postoperative anticoagulation, can lead to significant swelling (6, 10). This swelling poses challenges for prosthetic fitting and rehabilitation. Among the strategies to address this issue, DAA stands out as a particularly beneficial surgical technique for these patients. As a minimally invasive procedure performed through the intermuscular interval, DAA minimizes trauma to surrounding tissues, thereby reducing the risk of postoperative swelling. In addition to the advantages of DAA, postoperative care plays a critical role in managing residual limb swelling. The use of elastic bandages for wrapping the stump has proven effective in controlling swelling and promoting optimal healing (13).

In summary, DAA offers significant advantages for surgical procedures involving the residual limb. Unlike other techniques, DAA does not require additional fixation pins on the residual limb, allowing for easier manipulation during surgery. Its minimally invasive nature, combined with faster recovery and the absence of postoperative positioning restrictions, enables patients to engage in early joint mobilization without the risk of dislocation (8). Furthermore, the reduced trauma associated with DAA effectively minimizes postoperative swelling of the residual limb. These benefits make DAA particularly advantageous for patients with ipsilateral transfemoral amputation, as it addresses both surgical and rehabilitative challenges while optimizing outcomes.

## Conclusions

The management of total hip arthroplasty in patients with ipsilateral transfemoral amputation presents several challenges, including manipulation of the residual limb, exposure of the femoral side, and postoperative swelling. The direct anterior approach (DAA) allows for efficient surgical manipulation of the residual limb, while also minimizing postoperative swelling. Additionally, patients experience no positional restrictions and enjoy a faster recovery. Given these significant benefits, the minimally invasive DAA is a highly effective option for achieving optimal outcomes in such cases. However, further studies involving larger populations are needed to better evaluate the outcomes and validate the use of DAA in this setting.

## Data Availability

The original contributions presented in the study are included in the article/Supplementary Material, further inquiries can be directed to the corresponding author.
